# Preparation of capsaicin-loaded ultrafine fiber film and its application in the treatment of oral ulcers in rats

**DOI:** 10.1038/s41598-023-40375-3

**Published:** 2023-08-25

**Authors:** Xue Wang, Yu Xiong, Xinxin Zheng, Liang Zeng, Jinglin Chen, Lizhen Chen, Liping Zhong, Zhigang Liu, Jia Xu, Youhong Jin

**Affiliations:** 1 The Department of Periodontology, The Affiliated Stomatological Hospital of Nanchang University, Nanchang, 330006 Jiangxi People’s Republic of China; 2 The Key Laboratory of Oral Biomedicine, Nanchang, 330006 Jiangxi People’s Republic of China; 3 Jiangxi Province Clinical Research Center for Oral Diseases, Nanchang, 330006 Jiangxi People’s Republic of China; 4 Australian Rivers Institute and School of Environment and Science, Griffith University, Nathan, QLD 4111 Australia

**Keywords:** Materials science, Nanoscience and technology

## Abstract

A drug-loaded diaphragm is an easy-to-use and effective drug delivery system that is often used to treat mouth ulcers. In this study, an ultrafine fiber film loaded with capsaicin was successfully prepared using the electrospinning technology. poly-l-lactic acid and gelatin were selected as the matrix materials to form the composite fiber, and trifluoroethanol was used as a co-solvent for poly-l-lactic acid, gelatin and capsaicin to prepare the spinning solution, which was simple to fabricate. The prepared fiber films were characterized based on their microscopic morphology and tested to derive their mechanical properties. Thereafter, the capsaicin release behavior of the film was investigated. In vitro experiments revealed certain anti-inflammatory and antibacterial abilities while animal experiments revealed that the capsaicin-loaded ultrafine fiber film could promote the healing of oral ulcers in rats. Healing of the tongue tissue in rats administered 10% capsaicin-loaded fiber film was found to be better than that in rats administered the commercial dexamethasone patch. Overall, this development strategy may prove to be promising for the development of oral ulcer patch formulations.

## Introduction

Oral ulcer is a common disease of the oral mucosa that occurs in the lip mucosa, buccal mucosa, tongue, etc. Oral ulcer is excruciatingly painful during attacks, easily affects people's daily life, and has a certain recurrence, periodicity, and self-limitation ^1,2^. The etiology of oral ulcers has not been well studied; however, immunity, genetics, psycho-spirituality, vitamin deficiency, etc. may be factors that contribute to their occurrence. Owing to the uncertainty of its pathogenesis, the treatment methods for oral ulcer are diverse. Clinical treatment is usually associated with local symptomatic treatment^3–9^, and aims to reduce the inflammatory response, relieve pain, prevent deterioration of the ulcer surface, prevent secondary infection, promote healing, and reduce the attack duration. Local treatment usually involves anti-inflammatory and pain-relieving drugs, including steroids, antibiotics, etc. However, long-term use of drugs, such as antibiotics or steroids, may lead to osteoporosis, gastrointestinal irritation, bleeding tendency, and other side effects^3–9^^.^ Therefore, the search for alternative drugs with fewer side effects has garnered remarkable interest.

Capsicum has been consumed by humans as a spice ingredient for over 6000 years^10^, and similar to salt, is one of the most widely consumed condiments. Capsaicin (8-methyl-*N*-vanillyl-6-nonenamide) is the main active ingredient in red peppers^11^, and was first extracted from red peppers by Christian Friedrich Bucholz in 1816^12^. Capsaicin exhibits a variety of pharmacological activities, including analgesic^13^, anti-apoptotic^14^, anti-inflammatory^15^, anti-tumor^16^, anti-bacterial^17–19^, blood pressure regulation^20^, etc. At the beginning of the twenty-first century, Winston^21^ reported the use of capsaicin for pain treatment. According to the study, capsaicin was used for pain relief because of its binding with TRPV1 receptors distributed in class C primary afferent fibers, which selectively disrupt class C primary afferent fibers. Scheffler et al.^22^ conducted an 8-week double-blind controlled study to determine the effect of topical capsaicin cream on pain relief in diabetic neuropathy. Based on their findings, topical capsaicin (i.e., 0.075% w/v) was identified as a safe and effective treatment for diabetic neuropathic pain. Tang et al.^23^ showed that capsaicin inhibited lipopolysaccharide-induced inflammatory cytokine production by upregulating LXRα. Jiang et al.^24^ demonstrated that capsaicin significantly enhanced the re-epithelialization of ulcer tissue and promoted the healing of oral ulcers by reducing the expression of TNF-α and IL-6.

In addition to the drugs, the form of drug delivery is another key factor. The common drug dosage forms for oral ulcers include oral rinse, gels, films, etc.^25^ Due to the unique salivary environment and muscle movement characteristics of the mouth, films have many distinct advantages. In particular, films cover the wound surface to reduce external stimulation, significantly improve the targeting of drug delivery, help maintain the drug concentration on the wound surface, and prolong the drug delivery time. Therefore, the development of a drug-delivery membrane that can adhere to the oral mucosa and be easily fabricated is essential for clinical applications.

In this study, an ultrafine fiber film sheet loaded with capsaicin was prepared via the electrospinning technique^26,27^. The fiber matrix consisted of poly-l-lactic acid and gelatin, which have good biocompatibility. poly-l-lactic acid has good mechanical properties^28^ and is insoluble in water, which enables the maintenance of the structure of the fiber diaphragm in the salivary environment of the mouth. Gelatin has good hydrophilicity^29^, which allows the fibrous membrane to adhere well to wet wounds. The microstructure and morphology of the diaphragms were characterized, and their physicochemical properties were tested^30^. Further, the anti-inflammatory and antibacterial effects of the film were tested in vitro^31–34^ and the effectiveness of the film for the treatment of oral ulcers was evaluated using a rat model^24^.

## Materials and methods

### Materials

Poly-l-lactic acid (PLLA, Mv = 40,000, Zhejiang Hisun Biomaterials Co., Ltd); gelatin (Gel, porcine skin, type A, Sigma-Aldrich), lipopolysaccharide (LPS, Sigma-Aldrich); PBS buffer (1×, pH 7.2–7.4, Solarbio), Tween 80 (Solarbio), RPMI 1640 medium (Solarbio), penicillin (Solarbio), trypsin–EDTA mixture (Solarbio); phenol (Aladdin), fluorescein isothiocyanate isomer I (90%, Aladdin); capsaicin (Cap, HPLC ≥ 98%, Chengdu Desite Biotechnology Co., Ltd.); 2,2,2-trifluoroethanol (TFE, 99.8%, Shanghai Acmec Biochemical Co., Ltd); Zoletil 50 (Virbac, China); Xylazine (Sigma-Aldrich); Dexamethasone acelate oral mucoadhesive patches (DXM, Shenzhen Taitai Pharmaceutical Co., Ltd.); Cell Counting Kit-8assay kit (CCK8, Dalian Meilun Biotech Co., Ltd); fetal bovine serum (GIBCO); LB agar (GIBCO); LB agar medium (Qingdao Hi-tech Industrial Park Hope Bio-technology Co., Ltd), LB Broth (Qingdao Hi-tech Industrial Park Hope Bio-technology Co., Ltd.); mouse interleukin 6 (IL-6) kit (MM-0163M1, Jiangsu Enzyme Immune Industrial Co., Ltd.), mouse tumor necrosis factor a (TNF-α) kit (MM-0132M1, (Jiangsu Enzyme Immune Industrial Co., Ltd.); paraformaldehyde (4%, Biosharp); and mouse connective tissue L cell line clone 929 (Mouse fibroblast L929, Dalian Meilun Biotech Co., Ltd.) were employed in this study.

The bacteria, *Staphylococcus aureus* (*S. aureus*, ATCC25923) and *Escherichia coli* (*S. E. coli*, ATCC8739), were provided by Shanghai xuanya Biotechnology Co., Ltd.

All experimental protocols were approved by the Ethics Committee of Medical Laboratory Animals, Nanchang University. All rats were performed strictly under the Animal Management Rules of the Ministry of Health of the People's Republic of China and the guidelines for the Care and Use of Laboratory Animals (Document No. 2021121301). The authors complied with the ARRIVE guidelines. All efforts were made to minimize animal suffering and the number of animals used. 6 weeks old male SD rats (170–200 g) were provided by SPF (Beijing) Biotechnology Co., Ltd. The rats were housed under standard laboratory conditions, with a controlled temperature of 25 ± 1 °C for 24 h, relative humidity of 40–60%, and a light/dark photoperiod of 12 h. The animals had free access to food pellets and drinking water.

### Preparation of the PLLA-gelatin ultrafine fiber film

PLLA and gelatin were dissolved in TFE at different mass ratios to prepare a solution with a total mass volume ratio of 18%. The solution was stirred on a magnetic stirrer for 12 h to completely dissolve the solute. The electrospinning process was operated and performed in an electrospinning equipment (SS-1334, Beijing Ucalery Technology Development Co., Ltd). The solution was transferred to a syringe placed horizontally with a flat-headed needle (18 G) which was connected to the positive pole of the equipment. A flat sheet of aluminum foil, which was used as collector, was placed at a horizontal distance of 15 cm from the needle and was grounded. The voltage between the needle and collector was adjusted to 7 kV, and the syringe advance rate was 3.5 mL/h. After the solution was drawn out of the needle by an electric field force and flied to the collector, the fiber film was obtained on the flat aluminum foil, vacuum dried at 30 °C for 48 h and stored for further use.

### Preparation of the capsaicin-loaded ultrafine fiber film

Spinning solutions with capsaicin content of 0.1%, 0.5%, 1.0%, 2.5%, 5.0%, 10.0%, 17.0%, and 28.0% were prepared using TFE as a co-solvent for PLLA, gelatin and capsaicin, respectively. The total mass volume ratio of PLLA and gelatin was 18% (w/v) with the mass ratio of 1:1. The solution was stirred on a magnetic stirrer for 12 h to completely dissolve the solute. The spinning solutions were electrospun by the same process as above described separately and the collected films were vacuum dried at 30 °C for 48 h, and stored for further use.

### Morphology characterization

The prepared fiber film was cut into a 2 mm × 2 mm sample size strip, attached to a silicon wafer with conductive adhesive, and observed under a scanning electron microscope (SEM, JSM-6701F, JEOL, Japan) after gold sputtering coating for 90 s. After the voltage was applied, the magnification was adjusted to select the appropriate field of view for observation of the fiber film morphology. Photo-images were then taken and saved. Qualitative elemental analysis was performed on selected areas of the sample using energy dispersive X-ray spectroscopy (EDX). Image J software was used to analyze the fiber diameter in the photos.

### Fourier transform infrared spectroscopy (FTIR)

The prepared fiber film was cut into a 2 mm × 2 mm sample size strip and evaluated using an infrared spectrometer (FT/IR-4200, JASCO) with scanning range of 4000–500 cm^−1^.

### Mechanical properties

Four different mass ratios of the PLLA-gelatin fiber film were cut into 40 mm × 10 mm sized rectangle sheets, ensuring a neat cut at the edges of the fiber film. The universal material testing machine (CH17-621, Instron) parameters were set as follows: initial chuck distance, 10 mm; stretching speed, 5 mm/min; and tensile force, 4 N. Three replicates were set up for each sample of fiber film to assess the fiber film tensile performance.

### Release behavior of capsaicin

PBS (1×) containing Tween 80 (solubility enhancer for capsaicin) was used as the release medium (where VPBS:VTween80 = 99:1). 8 mg of capsaicin-loaded microfiber film was soaked in 20 mL of the release medium. The drug release experiments were carried out on a constant temperature shaker (speed 100 rpm) at 37 °C. 1 mL supernatant was aspirated at 15 min, 30 min, 45 min, 60 min, 90 min, 120 min, 240 min, 360 min, and 480 min, respectively. An equal volume of release medium was then added to replenish the media (except the last time). The liquid collected from each trial was cryopreserved for centralized detection.

A certain concentration of capsaicin solution was prepared with the release medium, and the maximum absorption wavelength of capsaicin was determined via scanning in the wavelength range of 200–400 nm using a UV–Vis spectrophotometer (V-750, JASCO) with the release medium as a reference.

Capsaicin solutions of 3.125 mg/L, 6.25 mg/L, 12.5 mg/L, 25 mg/L, 50 mg/L, and 100 mg/L were prepared as standard samples using the release medium. The release medium was used as a blank control. The absorbance of each concentration of the capsaicin solution was measured at the maximum absorption wavelength obtained in the previous step. Thereafter, the standard curve was plotted with the absorbance as the vertical coordinate and the concentration of capsaicin in the solution as the horizontal coordinate.

The concentration of capsaicin in each sample solution was calculated from the equation derived from the standard curve. Thereafter, the cumulative release was calculated, and the cumulative release curve was constructed.

### Cytotoxicity testing

L929 cells were selected to assess the cytotoxicity of the material (mPLLA:mGel = 3:1, 2:1, 1:1, 1:2). RPMI-1640 medium containing 10% fetal bovine serum was used. A cell culture incubator was set at 37 °C and 5% CO_2_. Cells at the logarithmic growth stage were inoculated in 96-well plates, and 5 × 10^3^ cells per well were used in both the experimental groups (with material) and control groups (without material). Wells containing 100 μL of medium alone served as the blank group, and 5 replicate wells were established for each group. After 12 h of incubation, each well of the experimental group was incubated with the corresponding material, which was cut into circular diaphragms of 5 mm diameter and irradiated with UV light on both sides for 1 h. After 24 h and 48 h of incubation, the supernatant and material were discarded and 100 μL of freshly prepared medium containing 10% CCK8 was added to each well and incubated for 1 h. The absorbance of each well was measured at 450 nm using a microplate reader. Thereafter, the relative cell growth rate (RGR) was calculated according to the following Eq. (1)^35^.

1 $$\mathrm{RGR}=\frac{{\mathrm{OD}}_{\mathrm{expriment}}-{\mathrm{OD}}_{\mathrm{blank}}}{{\mathrm{OD}}_{\mathrm{control}}-{\mathrm{OD}}_{\mathrm{blank}}}\times 100\mathrm{\%}.$$

OD stands for optical density.

### Treatment effect of capsaicin-loaded ultrafine fiber film sheets on L929 inflammatory cells

A control group (no inflammation, no material), LPS group (with inflammation), and experimental group (with both inflammation and material) were established. L929 cells in the logarithmic growth phase were inoculated in 96-well plates with 3 × 10^5^ cells per well and incubated in a cell culture incubator for 12 h. 1 μg/mL LPS was added to each well of the experimental group according to the above grouping and incubated for 12 h. Thereafter, 5 mm diameter capsaicin-loaded ultrafine fiber film sheets with different capsaicin loading capacities were added to each well of the experimental group, and the supernatant and material were discarded at days 0, 1, 3, and 5 of incubation and 100 μL of freshly prepared medium containing 10% CCK8 was added to each well and incubated for 1 h, respectively. The absorbance value of each well was measured at 450 nm using a microplate reader and the average survival rates of the cells were calculated.

### Enzyme-linked immunosorbent assay (ELISA)

After 3 days of cell culture according to the above grouping, the material was removed, the supernatant was aspirated, and the levels of IL-6 and TNF-α were detected via ELISA at 450 nm using an enzyme marker. The procedure was performed in strict accordance with the kit instructions.

### In vitro antibacterial experiments

Experimental groups: Control group (saline filter paper sheet), capsaicin-loaded ultrafine fiber film groups (capsaicin content was 0.1%, 0.5%, 1.0%, 2.5%, 5.0%, 10.0%, 17.0%, and 28.0%, respectively).

The slant medium was inoculated to activate *S. aureus* and *E. coli*, respectively. After 3 repetitions, the bacteria were inoculated in LB liquid medium, and placed in a constant temperature culture shaker (temperature, 37 °C; shaking speed, 120 rpm) for 24 h. The concentration of the bacterial solution was diluted to 0.5 MCF, and stored for later use. The LB nutrient agar was sterilized at 121 °C for 30 min and cooled to 50–60 °C. Thereafter, 2.0 μL of the *S. aureus* and *E. coli* solutions were added, and shaken gently and consistently. 15 mL of the agar medium was poured into the petri dishes containing the bacterial solution (i.e., 15–20 mL per dish), and allowed to solidify. The bacterial culture dish was labeled with the experimental date, strain name, drug name, etc. The drug film was placed on the corresponding position where the markings were made. The Oxford cups were placed vertically on the drug film, and gently pressed to reduce the gap with the drug film. Thereafter, 100 μL of saline was added to the Oxford cups. The samples were incubated in a constant temperature incubator for 18 h. Three replicate samples were set up for each group. The size of the inhibition circle was determined after 18 h of incubation by measuring the dimensions of the corresponding inhibition circle with a vernier caliper, and then using the formula (2) to calculate the width of the inhibition zone of the sample.

2 $$ {\text{H }} = \, \left( {{\text{D }} - {\text{ d}}} \right)/{2,} $$

H is the inhibition zone width (mm); D is the inhibition zone outer average diameter (mm); d is the model diameter (mm).

### In vivo animal experiments

After 60 male SD rats were acclimatized for one week, 48 were randomly selected and divided into 8 groups comprising 6 rats each. One group served as the control group (with tongue ulcers but no drug treatment), six groups served as the material groups (with tongue ulcers and administered 0%, 1%, 5%, 10%, 17%, and 28% capsaicin-loaded ultrafine fiber film), and the last group served as the dexamethasone acetate ulcer patch group (with tongue ulcers and dexamethasone acetate ulcer patch).

Preparation of the rat oral ulcer model: rats were anesthetized (15 mg/kg Zoletil 50 and 9 mg/kg Xylazine) and fixed to ensure full exposure of their tongues. Thereafter, 7 micro L phenol solution (99% phenol) was dropped onto a sheet of round filter paper with a diameter of 3 mm. The filter paper sheet was placed in the middle of the dorsum of the rat's tongue for 50 s. The filter paper sheet was then removed, and the residual phenol was removed via rinsing with saline and kept for 24 h. After rats in the material group and dexamethasone acetate ulcer patch group were anesthetized, the corresponding film sheets for each group were applied to the ulcers (all film sheets were cut into circular film sheets with a diameter of 5 mm, and the front and back sides were irradiated with UV for 30 min each) once per day for 30 min for 7 days. During this period, the body weight of rats and the morphological changes of the oral ulcers were observed daily in each group. On day 8 of the experiment, the rats were sacrificed by decapitation under anesthesia (15 mg/kg Zoletil 50 and 9 mg/kg Xylazine), and their oral mucosal ulcers with 2 mm of normal tissue at the junction were removed and fixed in 4% paraformaldehyde solution for 24 h. The morphology of the ulcers was observed after HE staining. Of the other 12 rats, 6 were left untreated, and the remaining 6 were used to establish the oral ulcer model. The 12 rats were sacrificed by decapitation under anesthesia (15 mg/kg Zoletil 50 and 9 mg/kg Xylazine). After 24 h. The oral mucosa of rats was retrieved from the dorsal middle of the tongue with 2 mm of normal tissue at the junction, fixed in 4% paraformaldehyde solution for 24 h, and stained with HE to observe and examine the histomorphology of the ulcer.

### Data analysis

All data were statistically analyzed using SPSS 22.0 statistical software. Statistical significance was defined as p < 0.05.

## Results and discussion

### Morphology of the fiber film

**Figure 1:**
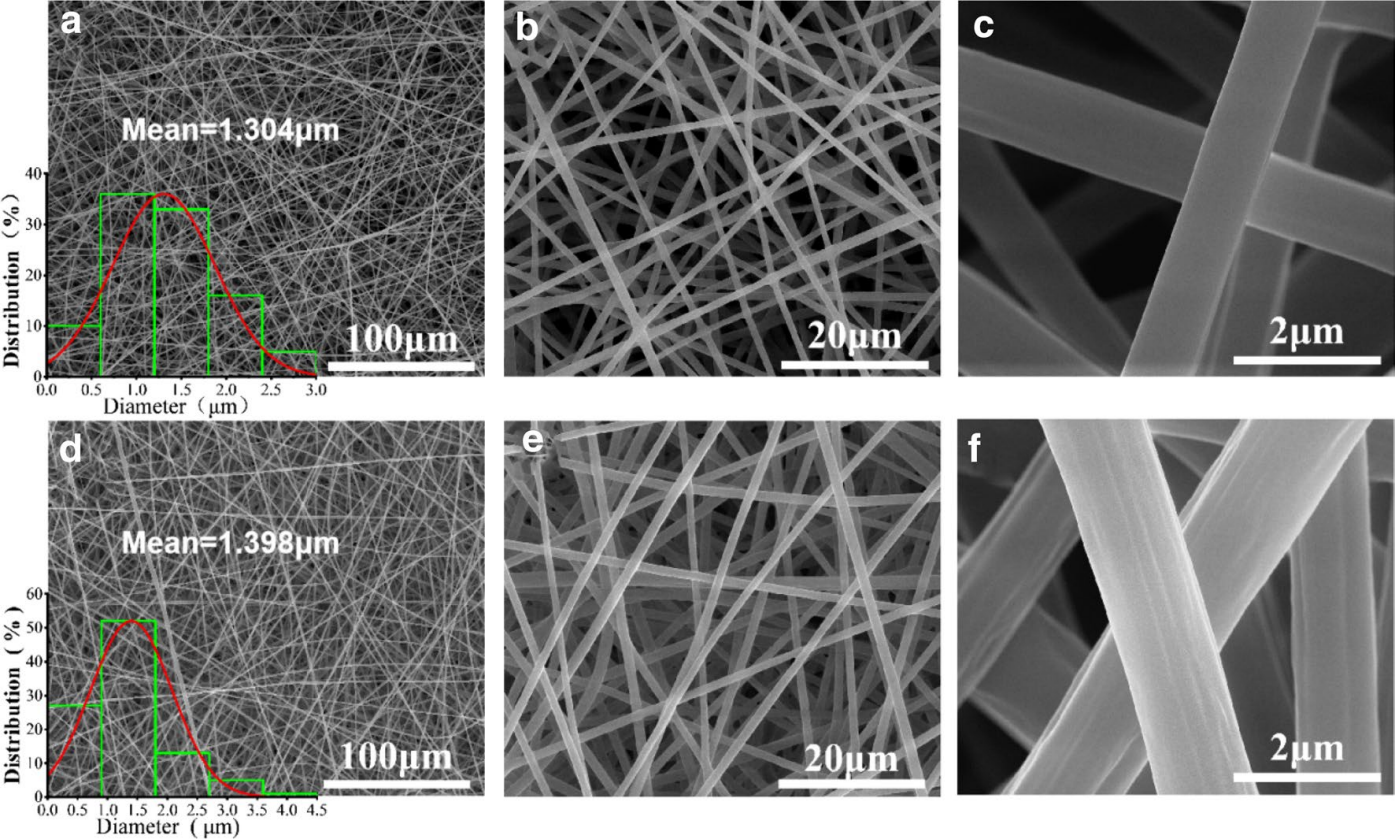
SEM images and fiber diameter distribution of PLLA-Gel fiber film (**a**–**c**) and capsaicin-loaded PLLA-Gel fiber film (**d**–**f**).

Figure 1a–c showed the SEM images of the PLLA-gelatin fiber film (where mPLLA:mGel = 1:1). Based on the figures, the PLLA-gelatin fiber film consisted of a large number of disordered ultrafine fibers stacked with an average fiber diameter of approximately 1.3 μm and a smooth fiber surface, and without beading structure, which indicates that the spinning liquid system used in this study had good spinnability. Figure 1d–f showed the SEM images of the capsaicin-loaded ultrafine fiber film (mPLLA:mGel = 1:1, 28% capsaicin). Notably, the capsaicin-loaded fibers appeared very similar to the non-capsaicin-loaded fibers in terms of diameter and morphology, which indicates that the addition of capsaicin did not significantly affect the fiber morphology.

**Figure 2:**
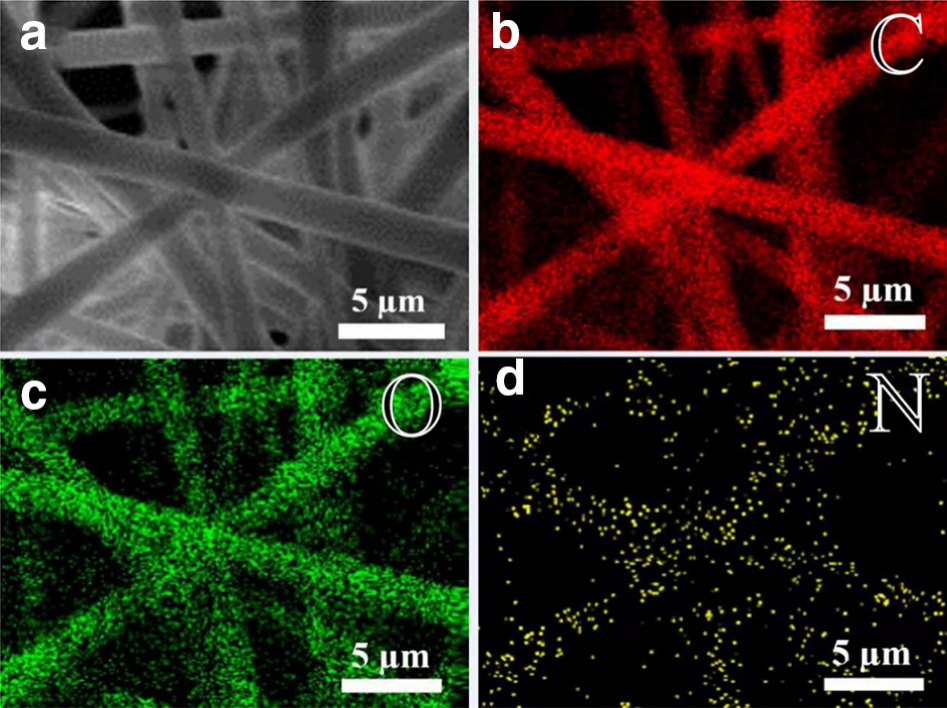
SEM (**a**) and EDX elemental mapping images of capsaicin-loaded PLLA fiber film; Red represents the presence of carbon (**b**); Green represents the presence of oxygen (**c**); Yellow represents the presence of nitrogen (**d**).

EDX is a commonly used method for the elemental analysis of materials. As capsaicin molecules contain nitrogen, EDX can be used to further demonstrate the distribution of capsaicin in fiber film. However, as gelatin molecules also contain nitrogen, poly-l-lactic acid fiber film loaded with capsaicin was prepared for EDX analysis to exclude the interference of nitrogen in gelatin molecules. Figure 2 showed the results of the elemental mapping of the capsaicin-loaded PLLA fiber film using EDX. The fibers were identified to contain nitrogen element (yellow colour) in addition to a large amount of carbon (red colour) and oxygen (green colour) elements, which confirms that capsaicin can be loaded into the fiber via the method employed in this study.

**Figure 3:**
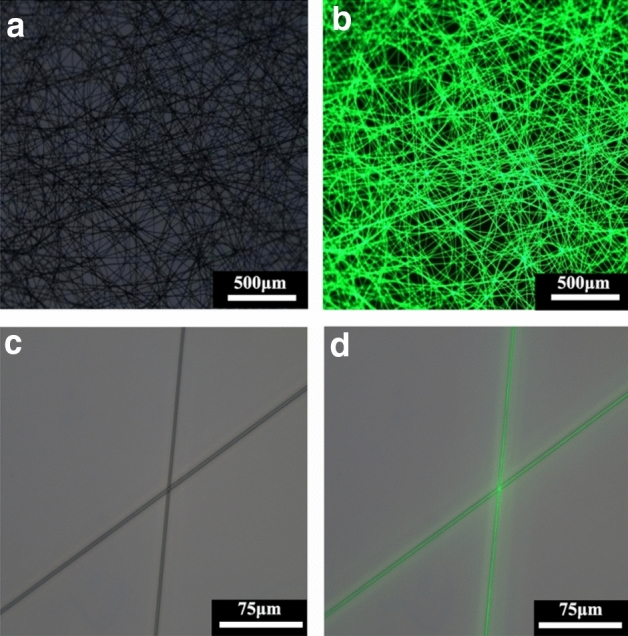
Fluorescence microscope images of fluorescent-loaded PLLA-Gel fiber film.

To reflect the loading of capsaicin in fibers more intuitively, fluorescein isothiocyanate (isomer I), which is also soluble in TFE, was used instead of capsaicin, and loaded into PLLA-gelatin fibers using the same method. Based on the fluorescence microscope results, each fiber could emit green fluorescence with uniform brightness under the excitation light (Fig. 3), which indicates successful loading of fluorescein into each fiber and even distribution. As both capsaicin and fluorescein are small molecules that are soluble in TFE, this result can be regarded as an indirect demonstration of the distribution of capsaicin in PLLA-gelatin fibers.

### FTIR

**Figure 4:**
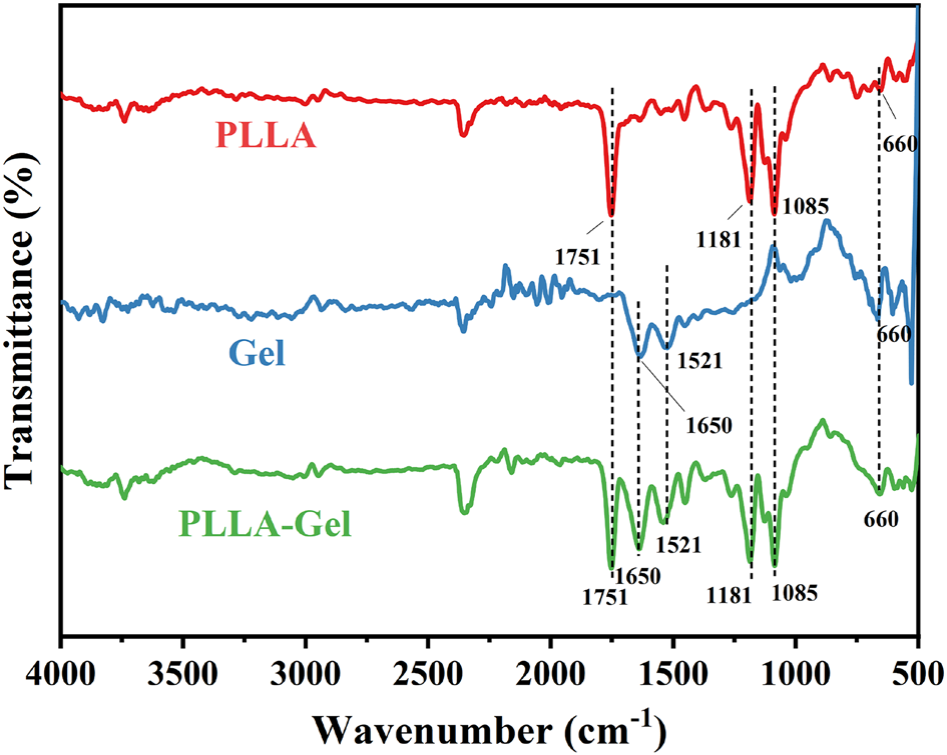
Infrared spectra for the PLLA (**a**), gelatin (**b**), and PLLA-gelatin (**c**) fiber films.

Figure 4 showed the infrared spectra for the PLLA fiber film, gelatin fiber film, and PLLA-gelatin fiber film. The PLLA fiber film was found to have obvious absorption peaks at 1751 cm^−1^, 1181 cm^−1^, and 1085 cm^−1^, which correspond to the characteristic peaks of the ester carbonyl and ether ester in the PLLA molecule. The gelatin fiber membrane had absorption peaks at 1650 cm^−1^ and 1521 cm^−1^, which correspond to the amide I band and amide II band in the gelatin molecule. In the spectrum for the PLLA-gelatin fiber film, absorption peaks were obtained at 1751 cm^−1^, 1181 cm^−1^, and 1085 cm^−1^ (indicating PLLA), and 1650 cm^−1^ and 1521 cm^−1^ (indicating gelatin). This result indicates that PLLA and gelatin can be successfully prepared into composite fiber film via the method employed in this study.

### Mechanical properties

**Figure 5:**
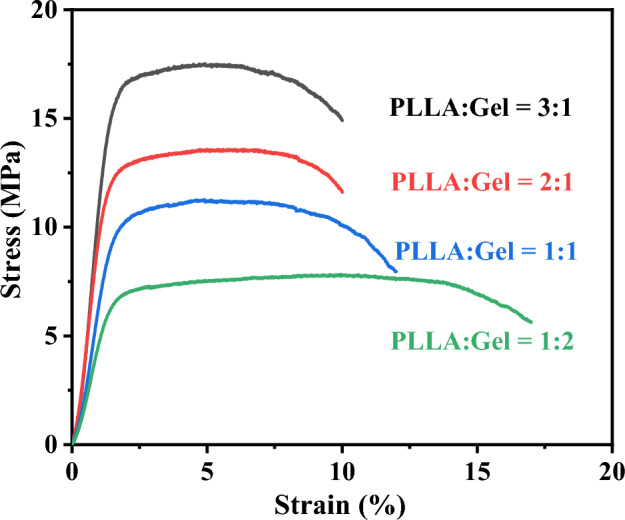
Stress–strain curve for the PLLA-gelatin fiber films at PLLA to gelatin mass ratios of 3:1, 2:1, 1:1, and 1:2, respectively.

Figure 5 showed the stress–strain curves for the ultrafine fiber film formed with different PLLA and gelatin mass ratios. It could be seen that with the relative content of PLLA in the fiber film decreases, the tensile strength and Young's modulus of the films decrease significantly, while the elongation at break increases. This may be because the mechanical properties of PLLA are better than gelatin. However, PLLA lacks hydrophilicity while gelatin has excellent hydrophilicity.

Considering both the hydrophobicity and mechanical properties of the material, the fiber film with a PLLA to gelatin mass ratio of 1:1, which satisfies the conditions for both hydrophilicity and mechanical strength, was selected for the subsequent experiments.

### Release behavior of capsaicin

**Figure 6:**
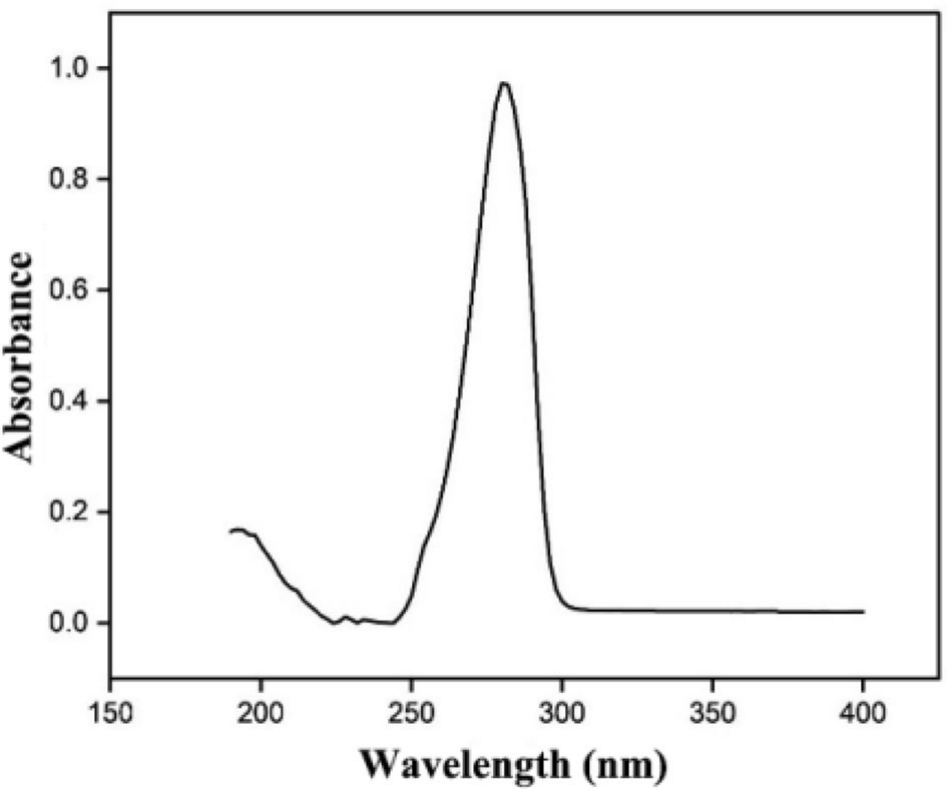
UV absorption spectrum for capsaicin.

As shown in Fig. 6, capsaicin was identified to have an obvious absorption peak at 280 nm. As a result, 280 nm was selected as the measurement wavelength for the determination of capsaicin content in the capsaicin-loaded microfiber membranes.

**Figure 7:**
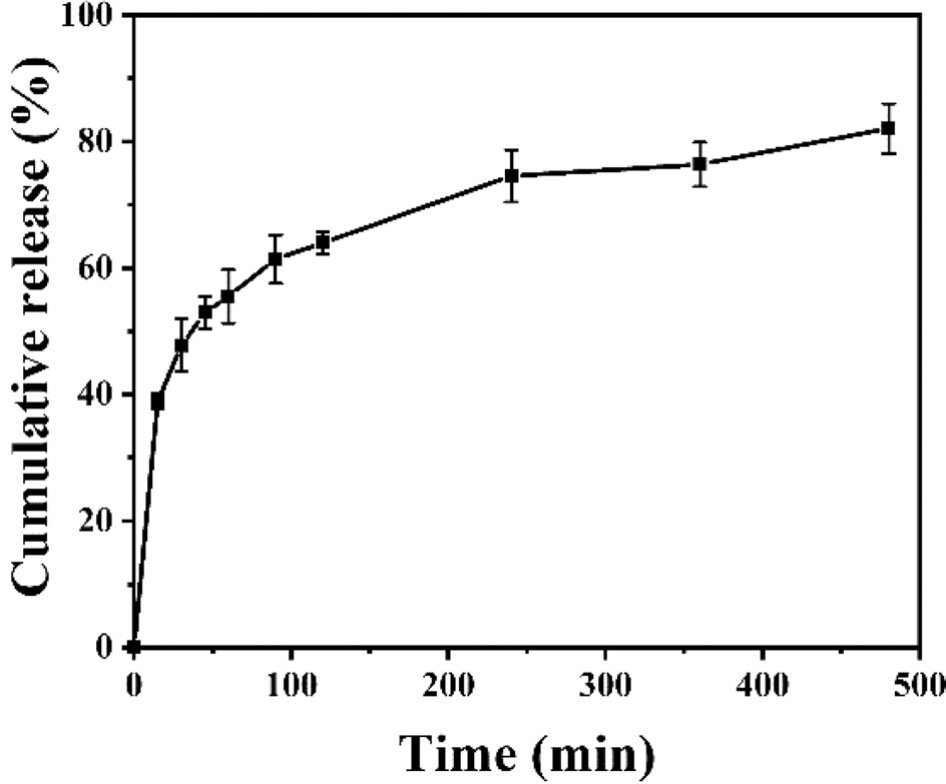
Cumulative drug release curve for capsaicin over an 8 h period.

Figure 7 showed the cumulative release curve for capsaicin in an aqueous release medium. The cumulative release of capsaicin rapidly reached 38.92% within 15 min and 61.39% by 90 min. Thereafter, the release curve for capsaicin displayed a significant slowing down trend and reached 82.05% at 8 h. This phenomenon may be due to the large specific surface area of the ultrafine fiber film, resulting in a larger proportion of capsaicin distributed on the surface of the material, which leads to a rapid initial release. This drug release behavior is beneficial for the treatment of oral ulcers^36^, where the drug-loaded film is applied to the wound, and rapid release of the drug at the early stage facilitates the quick relief of bouts of pain and other effects. The subsequent slow continuous release of the drug will then maintain its concentration at the wound.

### Cytotoxicity experiments with the PLLA-gelatin fiber films

**Figure 8:**
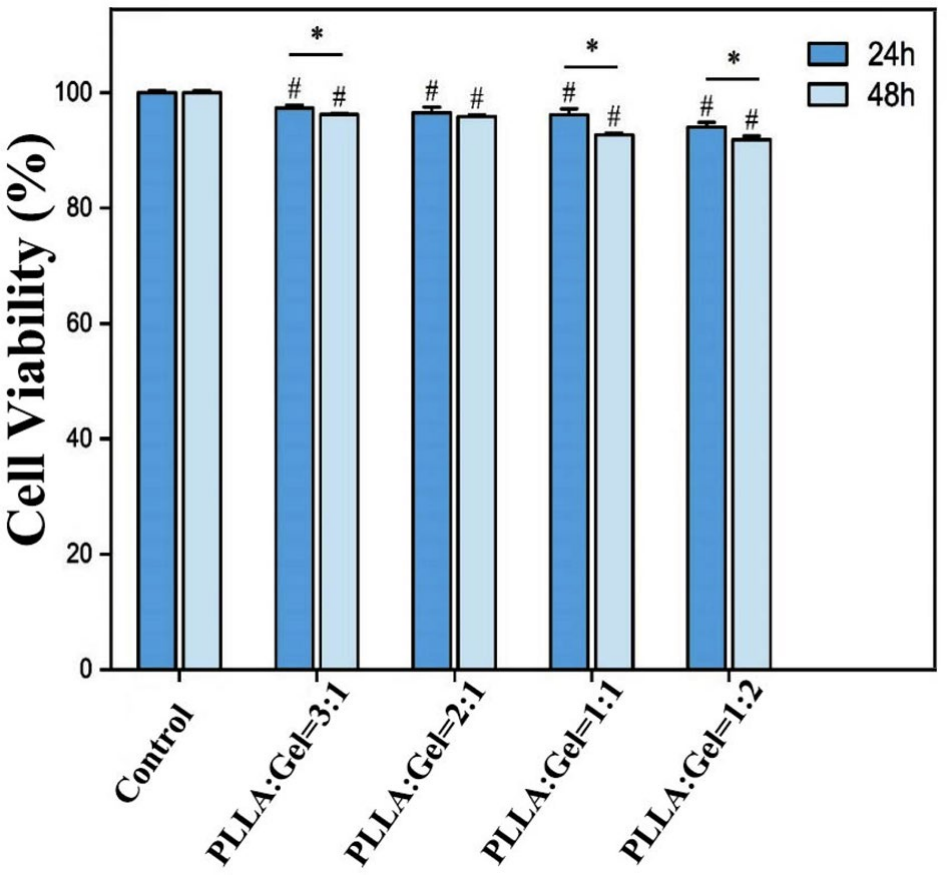
Cell viability for the PLLA-gelatin fiber films. *Denotes comparison between time points p < 0.05; ^#^Denotes comparison with the control group p < 0.05.

Figure 8 showed the cell survival rates after co-culture of the PLLA-gelatin (mPLLA:mGel = 3:1, 2:1, 1:1, 1:2) fibrils with L929 cells at different mass ratios for 24 h and 48 h. The cell survival rates varied slightly between groups at different incubation times; however, all groups still had cell survival rates exceeding 85%. This finding indicates that the polylactic acid-gelatin blended fibrous membrane sheet has minimal toxicity to L929 cells and can be used as a safe capsaicin loading material.

### Effect of the ultrafine fiber films with different capsaicin loading on the viability of L929 cells stimulated by LPS

**Figure 9:**
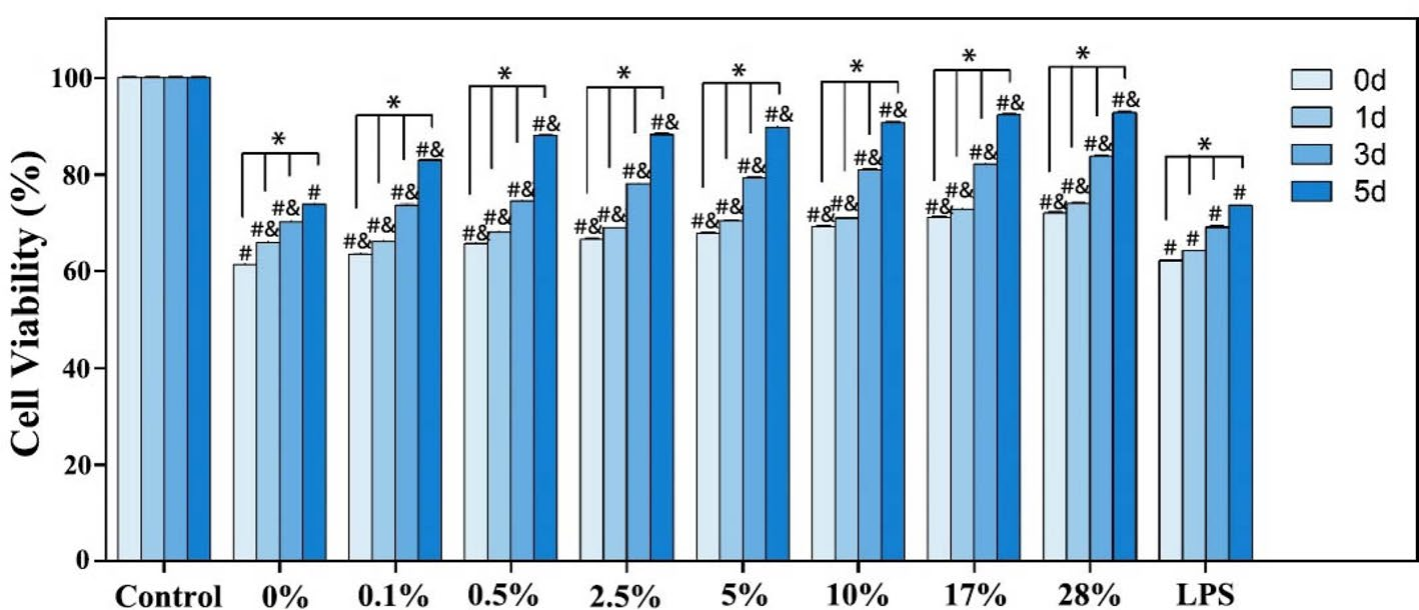
Effect of the capsaicin loaded ultrafine fiber films with different drug loading on the cell viability of L929 cells stimulated with LPS. *Denotes comparison between time points p < 0.05; ^#^Denotes comparison with the control group p < 0.05; ^&^Denotes comparison with group LPS p < 0.05.

Figure 9 showed the cell growth of fiber films with different capsaicin contents co-cultured with inflammatory L929 cells treated with LPS for 5 days. Cell viability was significantly lower in all groups relative to the control group at all time points. This result was mainly due to the role of LPS, which can cause cellular inflammation. Cell survival was significantly higher in the capsaicin loaded fiber film groups than in the LPS group, and the viability of L929 cells increased significantly as the drug loading and co-culture time increased. The above experimental results indicate that LPS could exhibit a strong growth inhibitory effect on L929 cells, and the inhibition rate could be as high as approximately 38%; however, the presence of capsaicin-loaded ultrafine fiber films could alleviate the growth inhibitory effect of LPS on L929 cells, and this alleviating effect was enhanced as drug loading increased. In fact, by day 5, the cell viability of the fiber film group with 28% drug loading recovered to 92.812%.

### ELISA to determine the effect of ultrafine fiber films with different capsaicin loading on the expression of IL-6 and TNF-α by LPS-stimulated L929 cells

**Figure 10:**
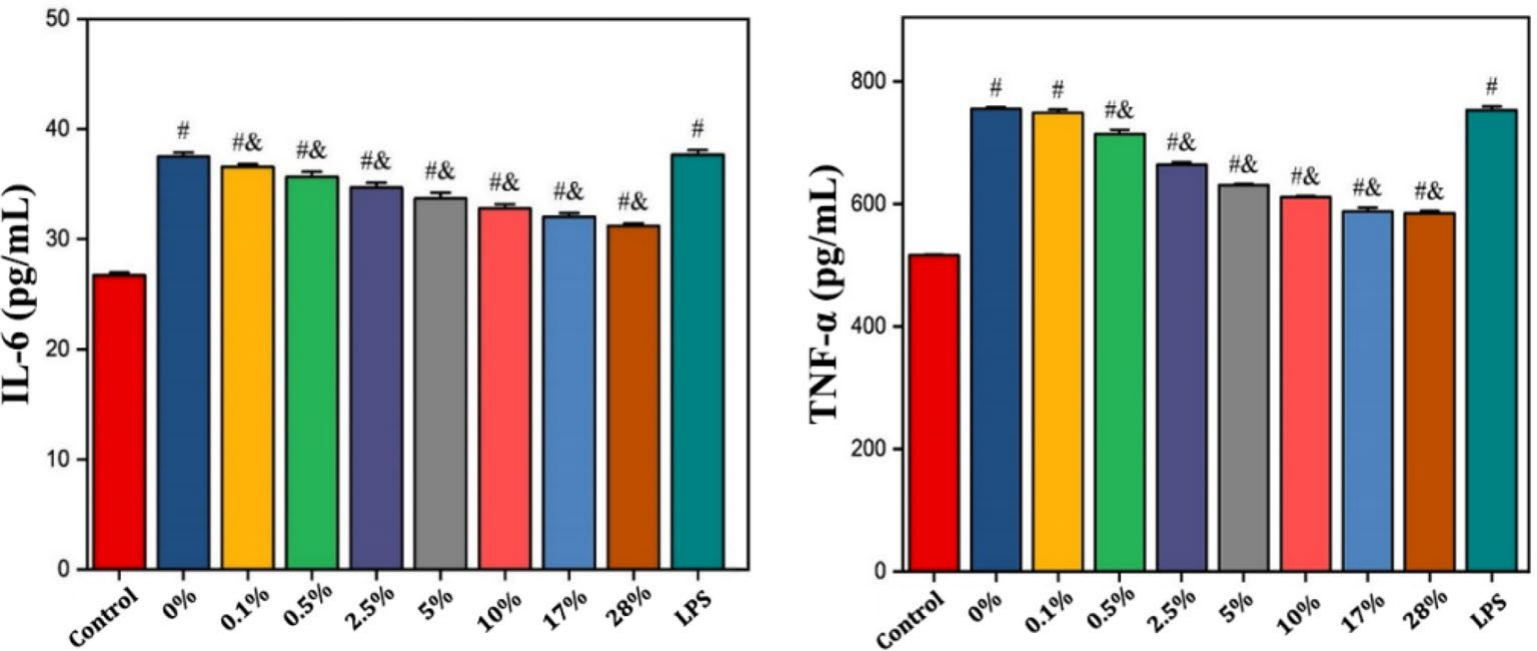
Effect of the capsaicin ultrafine fiber films with different drug loading on the expression of IL-6 and TNF-α by LPS-stimulated L929 cells. ^#^Indicates p < 0.05 compared to the control group; ^&^Indicates p < 0.05 compared to group LPS.

Figure 10 showed the IL-6 and TNF-α contents in the supernatant of the fiber films with different capsaicin contents after co-culture with inflammatory L929 cells treated with LPS for 3 days. The levels of IL-6 and TNF-α in all groups treated with LPS were significantly higher than those in the control groups, and the difference was statistically significant (p < 0.05), which indicated that L929 cells produced more inflammatory factors, such as IL-6 and TNF-α, under LPS stimulation. Compared to the LPS group, the expression of IL-6 was reduced in all capsaicin-loaded ultrafine fiber film groups, except the group with a capsaicin loading of 0.0%. Further, the reduction in IL-6 expression increased as the capsaicin content of the material increased. Similarly, compared with the LPS group, the expression of TNF-α was reduced in all capsaicin-loaded ultrafine fiber film groups, except for the capsaicin-loaded 0.0% group and the 0.1% group. The reduction in TNF-α expression also increased as the capsaicin content of the material increased. The above experimental results indicate that PLLA-gelatin fiber films with a certain capsaicin content can effectively inhibit the expression of the inflammatory factors, IL-6 and TNF-α, when co-cultured with inflammatory L929 cells. Notably, this inhibitory effect was enhanced with an increase in capsaicin content in the material.

### In vitro antibacterial experiments on capsaicin-loaded ultrafine fiber films

**Figure 11:**
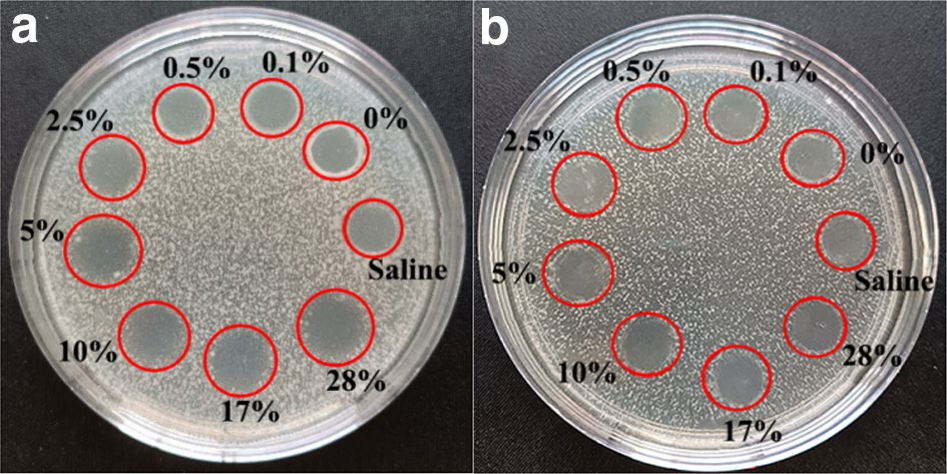
Antibacterial diagram for ultrafine fiber films with different capsaicin loading; (**a**) *Staphylococcus aureus*, (**b**) *Escherichia coli*.

Figure 11 showed the fiber films with different capsaicin contents after incubation with *S. aureus* and *E. coli*, respectively, for 18 h. The inhibition zone for *S. aureus* appeared in all material groups and the width of the inhibition zone increased as the capsaicin loading increased (Table 1). On the other hand, no inhibition effects were observed for any of the loading values for *E. coli*. Under the same incubation conditions, *S. aureus* was more significantly inhibited by capsaicin-loaded ultrafine fiber film than *E. coli*.

**Table 1:** Width (mm) of the capsaicin-loaded ultrafine fiber film inhibition zone.

Group	Saline	0%	0.1%	0.5%	2.5%	5%	10%	17%	28%
*S. aureus*	0	0.11 ± 0.06*	0.19 ± 0.01*	0.27 ± 0.11*	0.58 ± 0.25*	0.65 ± 0.17*	0.75 ± 0.22*	0.93 ± 0.12*	1.36 ± 0.26*
*E. coli*	0	0	0	0	0	0	0	0	0

*Differs significantly (p < 0.05) when compared against the saline group (negative control).

### General observation of the oral ulcer surface

**Figure 12:**
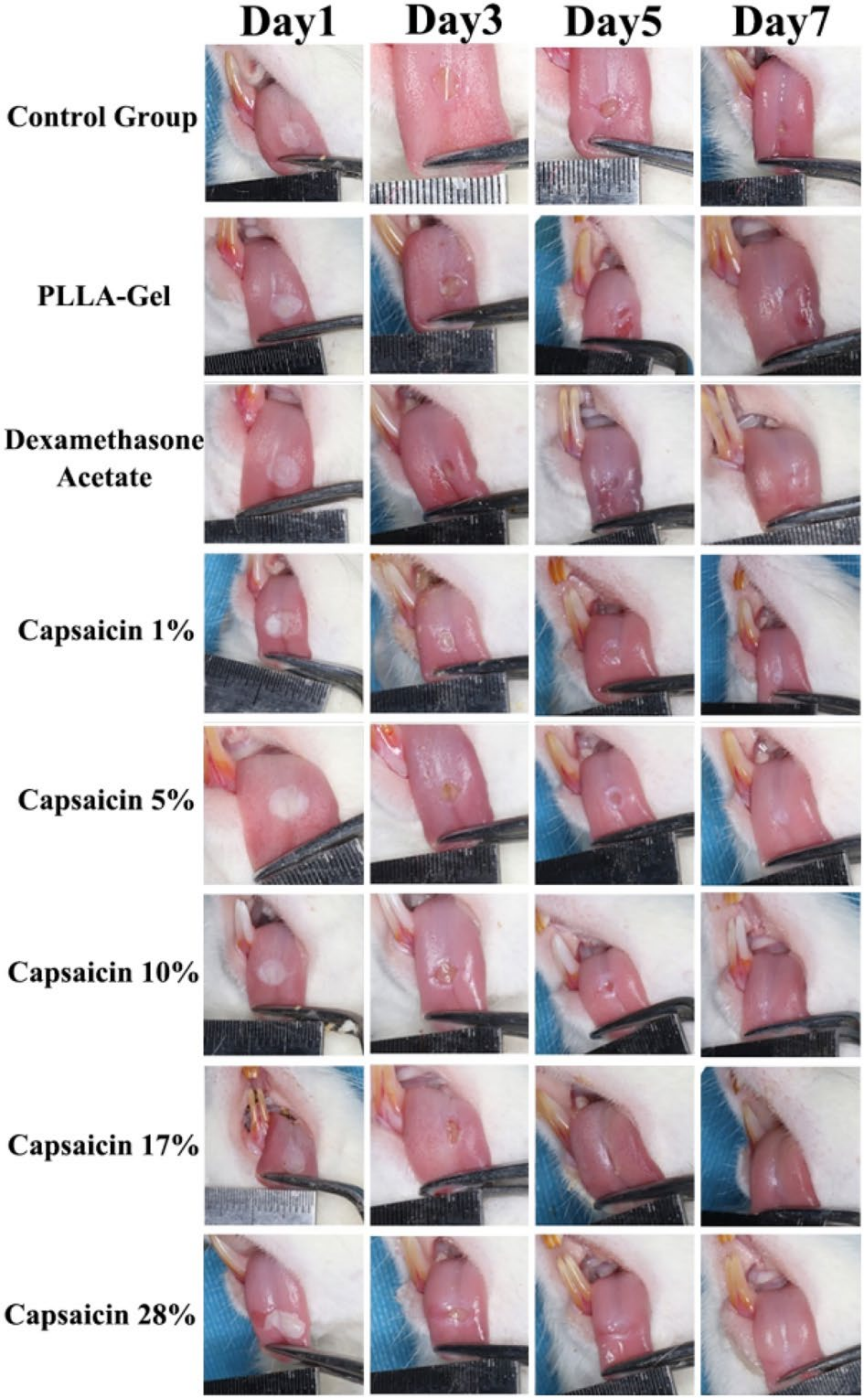
Effect of capsaicin-loaded ultrafine fiber films on oral ulcer model healing in rats.

As shown in Fig. 12 series Day 1, round ulcers with an approximate diameter of 3 mm were appeared on the tongue of all SD rats at 24 h after phenol treatment, which had a central depression and a white pseudomembrane covering the surface, and the surrounding mucosa appeared congested, redness and hyperaemia, and swelling. On day 5, most of the pseudomembranes had detached and the congestion and redness of the tissues gradually reduced. On day 7, the oral ulcers in the capsaicin loaded ultrafine fiber film and dexamethasone groups had almost healed and the surrounding mucous membranes were not congested and red. In the control group, the ulcers were still visible on the dorsal surface of the tongue and the tissue was depressed and edematous, but the symptoms were significantly reduced compared with those on day 1.

### HE staining of rat oral ulcers

**Figure 13:**
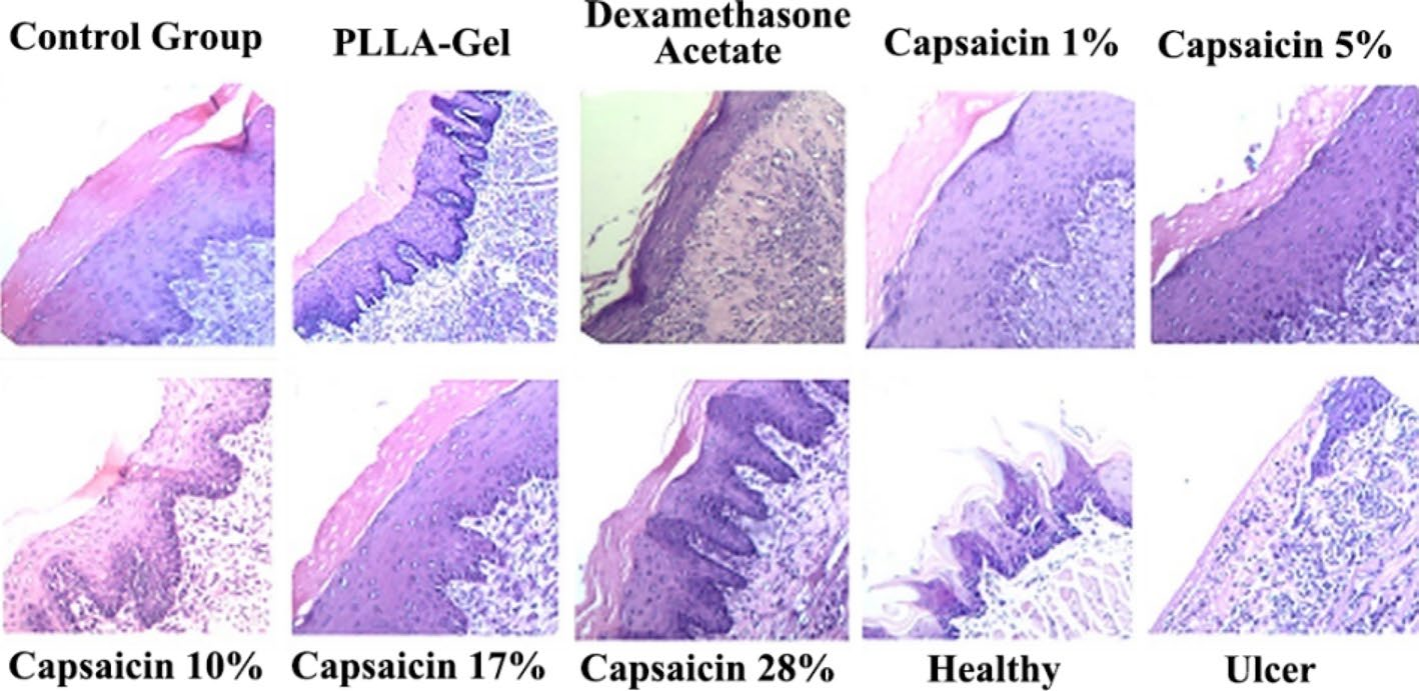
HE staining of the healing tissue for oral ulcers.

As shown in Fig. 13, the mucosal epithelium of rats in the healthy group (without creating ulcers) was intact, with connective tissue underneath and no obvious inflammatory cell infiltration. The oral mucosa of rats in the ulcer model group (tissues collected 24 h after the establishment of ulcers) was broken, the entire epithelial tissue had detached, and numerous inflammatory cells infiltrated the ulcers.

Compared with the healthy group and ulcer model group, the epithelium of the control group was slightly atrophied and some inflammatory cells, small blood vessels, and fibroblasts were visible, indicating that the oral ulcer was not completely healed. The PLLA-Gel group had prolonged epithelial pegs in the oral mucosa and the shape of filiform papillae disappeared. Further, the epithelial tissue of the dexamethasone group was slightly thinned, the blood vessels were slightly dilated and congested, and the underlying connective tissue was slightly edematous. The 1% capsaicin-loaded ultrafine fiber film group showed excessive non-keratinization of the epithelium, elongation of the epithelial pegs, partial disappearance of filiform papillae, and visible focal infiltration of inflammatory cells; 5% capsaicin-loaded ultrafine fiber film group showed that epithelium was slightly atrophic, the epithelial pegs were not prolonged, no inflammatory cells were observed, and the number of fibroblasts had increased. Notably, the 10% capsaicin-loaded ultrafine fiber film group showed that epithelium did not thicken, the epithelial pegs were not prolonged, no inflammatory cells were observed, the number of fibroblasts had increased, and the epithelial layer was close to that of normal mucosal tissue. The 17% capsaicin-loaded ultrafine fiber film group showed that epithelium was slightly atrophied and the number of fibroblasts had increased. Finally, the 28% capsaicin-loaded ultrafine fiber film group showed that epithelium was slightly atrophied and fibrous connective tissue was partially proliferated.

Compared with the control group and PLLA-Gel group, the mucosal tissue of each group administered the capsaicin-loaded ultrafine fiber film and the dexamethasone group displayed a large amount of fibroblastic tissue proliferation, indicating healing of the ulcer tissue, with a small amount of inflammatory cells visible in the low concentration drug group and a slight epithelial atrophy in the high concentration drug group. However, the tissue in the fiber film group with 10% drug loading was closest to normal mucosal tissue. This result indicates that capsaicin-loaded ultrafine fiber film has a positive effect on the treatment of oral ulcers in rats. In particular, when the capsaicin content was 10%, better healing of the oral ulcers was achieved than with the commercial dexamethasone patch. However, further studies are needed to elucidate the molecular mechanism whereby capsaicin promotes wound healing of oral ulcers.

## Conclusions

In this study, capsaicin-loaded ultrafine fiber films were prepared by using the electrospinning technique with PLLA and gelatin as the carrier materials. In vitro experiments confirmed that the capsaicin-loaded ultrafine fiber films can reduce the expression of TNF-α and IL-6 in inflammation models and have certain anti-inflammatory effects, which are enhanced with the increase in capsaicin content. In vitro antibacterial experiments confirmed the antibacterial effect of capsaicin-loaded ultrafine fiber films on *S. aureus*, and revealed enhancements with an increase in capsaicin content. The results of the animal experiments showed that the capsaicin-loaded ultrafine fiber film promoted the healing of oral ulcers in rats. Further, it was found that the ulcer healing of rats treated with 10% Capsaicin loading was better than other capsaicin-loading groups and the commercial dexamethasone patch. This study provides a new approach for the development of an oral ulcer patch formulation that can be used in clinical applications.

## Data Availability

All data generated or analyzed during this study are included in this article.
